# Radiation and Immunotherapy in Upper Gastrointestinal Cancers: The Current State of Play

**DOI:** 10.3390/ijms22031071

**Published:** 2021-01-22

**Authors:** Noel E. Donlon, Robert Power, Conall Hayes, Maria Davern, John V. Reynolds, Joanne Lysaght

**Affiliations:** 1 Department of Surgery, School of Medicine, Trinity College Dublin, Dublin 8, Ireland; donlonn@tcd.ie (N.E.D.); powerr8@tcd.ie (R.P.); chayes5@tcd.ie (C.H.); davernma@tcd.ie (M.D.); reynoldsjv@stjames.ie (J.V.R.); 2 Trinity St James’ Cancer Institute, St James’s Hospital Dublin, Dublin 8, Ireland

**Keywords:** radiotherapy, immunotherapy, dosing, timing, curative, upper gastrointestinal cancers

## Abstract

Radiotherapy remains one of the contemporary cornerstones of cancer treatment in the neoadjuvant, curative, adjuvant and palliative settings, either in isolation or as a multimodal approach. Moreover, recent advances in targeted immune checkpoint therapy have firmly established immunotherapy as the fourth pillar in cancer therapy alongside surgery, chemotherapy and notably radiotherapy. There is emerging evidence to suggest both radioresistance and reduced efficacy of immune checkpoint blockade (ICB) are potentiated by the tumour microenvironment (TME) and in fact modulating aspects of this immunosuppressive milieu is instrumental to unlocking anti-tumour immunity. The response rates of Upper Gastrointestinal (UGI) malignancies to ICB remains modest at 10–15%, compared to melanoma at 20–40%. Harnessing the effects of radiotherapy through remodelling of the TME using ICB as a radiosensitisor is an avenue showing promise. Here we explore the rationale behind combining radiotherapy with ICB, as a symbiotic relationship in shifting the balance in favour of anti-tumour immunity. We discuss the effects of radiotherapy on immunogenic cell death, the concept of the abscopal effect, the importance of the cGAS STING pathway, and their relevance in the context of the tumour microenvironment. Furthermore, dosing and timing of radiotherapy and ICB is now being evaluated for its synergistic effects on host tumour immunity, and we review the ongoing efforts and current available literature for single agent and dual agent ICB in combination multimodal therapy for both locally advanced operable and metastatic disease of the upper gastrointestinal tract.

## 1. Introduction

After years of effort to harness the immune system for the treatment of cancer, the advent of antibodies which target ‘immune checkpoints’, including programmed cell death protein 1 (PD-1) and cytotoxic lymphocyte antigen 4 (CTLA-4), has increased interest in immunological aspects of conventional therapies. These immune checkpoint inhibitors (ICIs), including pembrolizumab, tislelizumab, nivolumab (anti PD-1) and ipilimumab (anti CTLA-4), have led to dramatic and durable clinical responses in diverse cancers [1]. Cancers of the upper gastrointestinal tract (UGI) are common globally and account for a disproportionately high incidence of cancer-related mortality. In 2019, UGI malignancies accounted for 9% of cancer diagnoses and 13.5% of cancer related deaths worldwide [2]. The response rates for clinically approved ICIs in melanoma and non-small cell lung cancer are approximately 20–40% [2]; however, in UGI cancers, this decreases to 10–15% and ICIs are therefore largely confined to salvage treatment of advanced disease, with its use in the neoadjuvant and curative setting confined to select cases [3].

**Figure 1:**
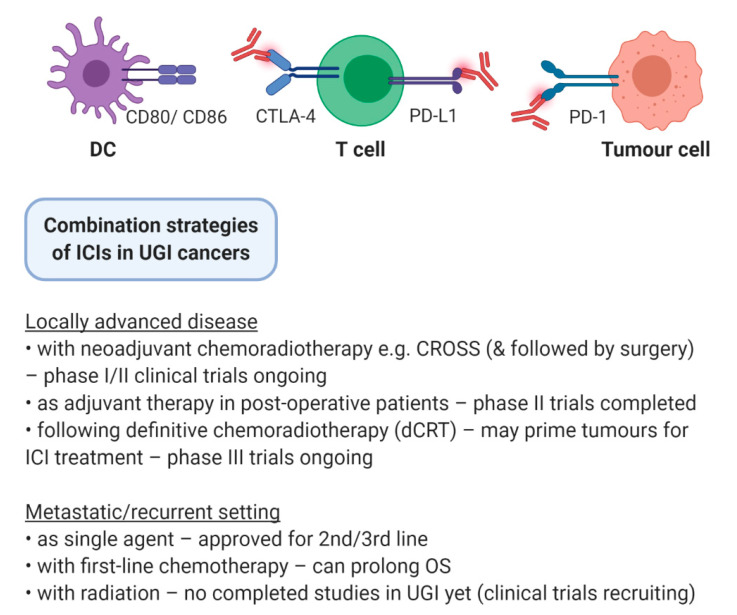
Combination strategies of ICIs in UGI cancers. Immune checkpoint inhibitors (ICIs) are under investigation in a variety of settings in upper gastrointestinal (UGI) cancers, including in conjunction with surgery, chemotherapy, radiation therapy, and multimodal combinations.

Radiotherapy has been one of the pillars for the management of neoplastic burden in cancer patients for over a century. It is used as a treatment modality in approximately 50% of cancer patients in the neoadjuvant, adjuvant, curative or palliative settings [4]. Tri-modality treatment of surgery, chemotherapy and radiotherapy is the standard of care in oesophageal adenocarcinoma (OAC) and oesophageal squamous cell carcinoma (OSCC), while in gastric cancer (GC) adjuvant chemotherapy use depends on surgical margins and the extent of lymph node dissection [5]. Radiation triggers DNA damage-induced cell death in cancer cells but can also modify the antigenicity and the adjuvanticity of tumours. This is by activating cytosolic DNA sensors, inducing immunogenic cell death, enhancing neoantigen expression and modulating the tumour microenvironment (TME) [6]. Therefore, combining immuno-oncology approaches with radiation could boost response to ICI in UGI cancers (Figure 1). In this review, we describe the mechanistic rationale for combining immunotherapy and radiotherapy, clinical trials of radio-immunotherapy in gastroesophageal malignancies and current strategies to optimise radiation regimen efficacy while minimising toxicity.

## 2. Mechanisms of Synergy

### 2.1. The Abscopal Effect

The “abscopal effect”, first described in 1953 by Mole, refers to regression of metastases outside the primary radiation field post irradiation [6]. This is not frequently observed in tumours treated with radiotherapy alone; a phase II trial of 60 patients with head and neck cancer did not report any abscopal responses as a secondary endpoint [7]. However, it appears abscopal response appears to occur more commonly when ICIs is used alongside radiotherapy, both in experimental models and clinical studies. In a mouse model of melanoma, CTLA-4 blockade alongside hypofractionated radiotherapy led to an abscopal effect [8]. Radiotherapy and PD-1 blockade has seen abscopal responses in mouse models of melanoma, renal cell carcinoma and thoracic cancers [9]. This has also been reported clinically, most prominently in melanoma patients treated with PD-1 blockade [10]. A pooled analysis of two clinical trials of ICIs in non-small cell lung cancer, found that the additional of radiotherapy increased the response rate of unirradiated lesions, and was associated with prolonged survival—suggesting that this phenomenon can confer a clinical benefit [11]. The mechanism of the abscopal effect is not clear, and relies on mostly preclinical data, but it is hypothesised that radiation induced cell death can release tumour antigens from the primary lesion. These antigens may be taken up by antigen-presenting cells (APCs), which migrate to lymph nodes to prime naïve CD4^+^ and CD8^+^ T cells [12]. The activated CD8^+^ cytotoxic T cells then travel to both the primary irradiated tumour and the non-irradiated metastatic site where cognate tumour antigens are recognised, and this can trigger immune-mediated elimination of malignant cells.

### 2.2. The Cyclic GMP-AMP Synthase-Stimulator of Interferon Genes (cGAS-STING) Pathway and Interferon Production 

Ionising radiation causes DNA damage directly, as well as indirectly through the formation of reactive oxygen species (ROS) that form double-stranded DNA breaks [13]. DNA released following radiation-induced cell death can activate the cytoplasmic DNA sensing cyclic GMP-AMP synthase (cGAS)-stimulator of interferon genes (STING) pathway [14]. DNA binds to cGAS, forming 2,3-cGAMP, which then activates STING. STING acts through two main pathways: (1) upregulation of type I interferon (IFN) release by activating IRF3 and by activating NF-κB, mainly by tumour infiltrating CD141^+^ dendritic cells, a subtype specialised in antigen cross-presentation [15]. (2) Type I interferons mediate recruitment and the effector function of CD8^+^ T cells [13]. Through these mechanisms, exogenous cGAMP and STING agonists enhance the efficacy of radiation in preclinical models.

Although triggering of type I IFN production by cGAS was well characterised, it was unknown how radiotherapy-induced this response until relatively recently. Classically it was thought that cGAS recognised cytosolic DNA, but the revised model indicates that DNA recognition occurs within cGAS containing micronuclei [16]. These micronuclei form when cells progress to mitosis, following DNA damage, and this explains the delayed onset between radiotherapy and innate immune signalling [17]. The cellular compartment responsible for mediating STING’s effects is unclear. Some models indicate that tumour cell-intrinsic STING is necessary, whereas others indicate tumour cell-derived DNA in exosomes contributes to radiotherapeutic immune responses [18,19]. The role of STING in radiation-induced immunity is controversial, as other studies indicate that STING activation may be tolerogenic by modifying the microenvironment and recruitment of myeloid-derived suppressor cells (MDSCs) [20]. Therefore, more work is needed to further elucidate the biology of the STING pathway in this context.

Type I interferons themselves have opposing effects on tumour and immune cells, stimulating the anti-tumour immune response while promoting tumour cell survival. Prolonged IFN signalling can induce a chronic immunosuppressive state, leading to the selection of radioresistant tumour clones [21]. A pre-existing high expression of interferon-stimulated genes (ISGs) in tumours is linked to resistance to radiation, chemotherapy and ICIs; potentially mediated by autocrine or paracrine tumour cell IFN signalling [22]. Across cancer types, patients with tumour infiltrating dysfunctional T cells show upregulated ISGs including T cell inhibitory ligands (e.g., PD-L1) and enzymes that inactivate granzyme B [23,24]. This suggests that combining ICI and radiation may only benefit those with low ISG signatures and could be detrimental in those with chronic interferon driven basal ISG phenotype.

### 2.3. Immunogenic Cell Death

**Figure 2:**
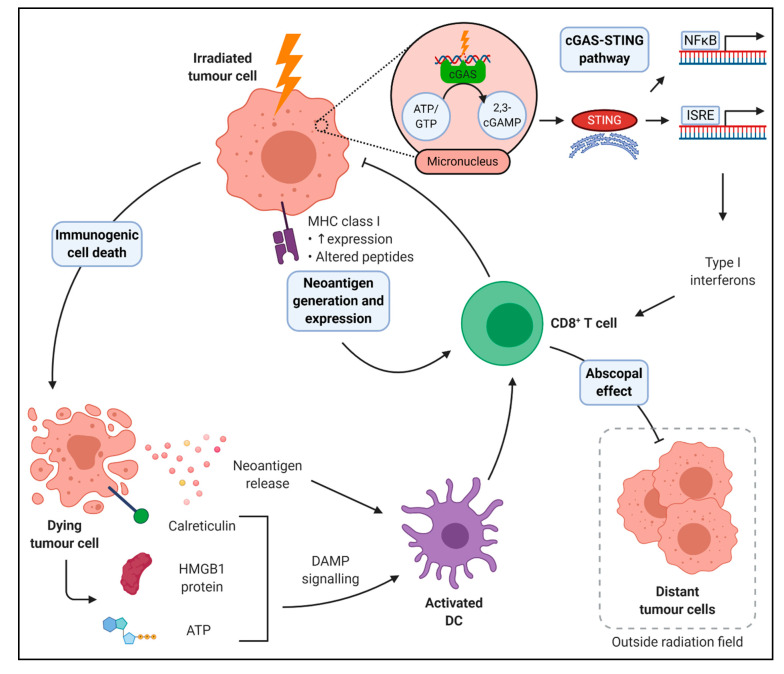
Immunogenic effects of radiation therapy. Radiation can augment anti-tumour immunity in several ways. Damage and death of cancer cells leads to release of tumour neoantigens and damage-associated molecular patterns (DAMPs) such as calreticulin, high motility group box 1 (HMGB1) protein, and ATP which activate dendritic cells (DCs) to prime and stimulate CD8^+^ T cells responsible for cancer cell detection and elimination. Moreover, radiation can increase the expression and alter the array of peptides presented on major histocompatibility (MHC) class I proteins, which CD8^+^ T cells use to identify transformed cells. Radiation-induced DNA damage can activate the cGAS-STING pathway leading to type I interferon production which enhances CD8^+^ T cell activity. Beyond local effects, radiation may induce systemic anti-tumour immunity if tumour-specific CD8^+^ T cells migrate to metastatic lesions; regression of distant tumour cells outside the radiation field is known as the abscopal effect.

Radiotherapy can augment the adjuvanticity of tumours through induction of immunogenic cell death. This promotes tumour antigen processing by the release of damage-associated molecular patterns (DAMPs) and activating necrotic or apoptotic pathways [25]. These DAMPs include calreticulin, ATP and HMGB1, and all are increased by radiotherapy. Calreticulin acts as a prophagocytic signal and opposes the survival signal of CD47 [26]. HMGB1 activates TLR4 and promotes antigen cross-presentation by blocking degradation of phagosomes [27]. ATP released into the TME binds to the P2X7 purinergic receptor on antigen-presenting cells. This activates the NLRP3 inflammasome, releasing IL-1β, which is essential for priming of cytotoxic T cells [28]. Therefore, the precise delivery of radiotherapy can convert a tumour into an in-situ vaccine, whereby neoantigens are released, and DAMPs enable efficient antigen presentation and effector immune cell function (Figure 2).

### 2.4. Neoantigen Generation and Expression

Elimination of tumour cells by cytotoxic T cells requires antigen presentation on MHC-I molecules. An accumulating body of evidence suggests that the tumour mutational burden and neoantigen load predicts clinical response to ICIs [29]. Radiation increases tumour cell MHC-I expression [30] and radiotherapy also expands the intracellular peptide pool, altering cellular MHC-I associated peptide profiles while upregulating presentation of existing peptides [31,32]. Radiation-induced DNA damage activates a cellular stress response, promoting transcription and expression of neoantigens [33]. In Non-Small Cell Lung Cancer (NSCLC), the *KPNA2* gene, a member of the nuclear transporter family, is involved in the nucleocytoplasmic transport pathway of a variety of tumour-associated proteins. Its expression is upregulated by radiation, and peptide fragments trigger activation and IFN production in the patients CD8^+^ T cells, therefore radiotherapy increases presentation of existing neoantigens, encouraging a CD8^+^ T cell response [33].

Neoantigens can be divided into clonal neoantigens, present in all tumour cells and are potent drivers of anti-tumour immunity, and subclonal neoantigens which are only present in a subset of tumours cells and are less immunogenic [29]. There is a concern that even if radiotherapy induced DNA damage elicits an effective antigen specific response it will only kill a small subset of tumour cells leaving the bulk of the tumour cells behind. Preclinical models suggest that radiotherapy in combination with ICIs is associated with increased diversity of the TCR repertoire. However, these tumours are dominated by a small number of high frequency T cells clones, suggesting an immune response is mounted against just a few clonal neoantigens [34]. Additional clinical data are needed to determine the contribution of radiation-created neoantigens in the anti-tumour response.

### 2.5. Remodelling of the Tumour Microenvironment

The mass of non-malignant cells and stromal tissue surrounding cancerous cells is referred to as the tumour microenvironment (TME), which is composed of numerous cell types including cancer-associated fibroblasts, endothelial cells, pericytes and a wide range of innate and adaptive immune cells [35]. Radiotherapy promotes a chemokine milieu amenable to T cell infiltration, including the secretion of CXCL16, which binds to CXCR1 on TH_1_ cells and activates CD8^+^ T cells [36]. In murine lung models, a combination of radiation and Ataxia Telangiectasia Mutated and RAD3 (ATR) inhibition promotes transcription of CXCL10, which binds to the immunostimulatory CXCR3 receptor on T cells [30]. Radiation also upregulates ICAM-1 and NKG2D ligand RAE-1γ (encoded by *Rae1g)* in vivo [37]. Once T cells have infiltrated a tumour, MHC-I, ICAM-1, RAE-1γ and NKG2D promote T cell arrest, tumour cell engagement and were found to be essential for the efficacy of a combination of radiotherapy and CTLA-4 blockade in mice [37]. Radiation also increases production of CCL5, which acts to recruit pro-inflammatory CCL2^+^CCL5^+^ macrophages both inside the tumour and in the peripheral circulation [38]. Low dose radiation promotes vascular renormalisation, which could be useful in immune-excluded tumours where stromal elements prevent effector immune cells from accessing the tumour parenchyma [39]. Radiation can also promote polarisation of M2-macrophages to an M1 pro-inflammatory phenotype [39]. M1-macrophages secrete TH_1_ cytokines (IFN-y, IL-12) and enhance CD8^+^ T cell activity. These M1-macrophage express high levels of inducible nitric oxide synthase (iNOS+) allowing nitric oxide dependent vessel normalisation [40].

**Figure 3:**
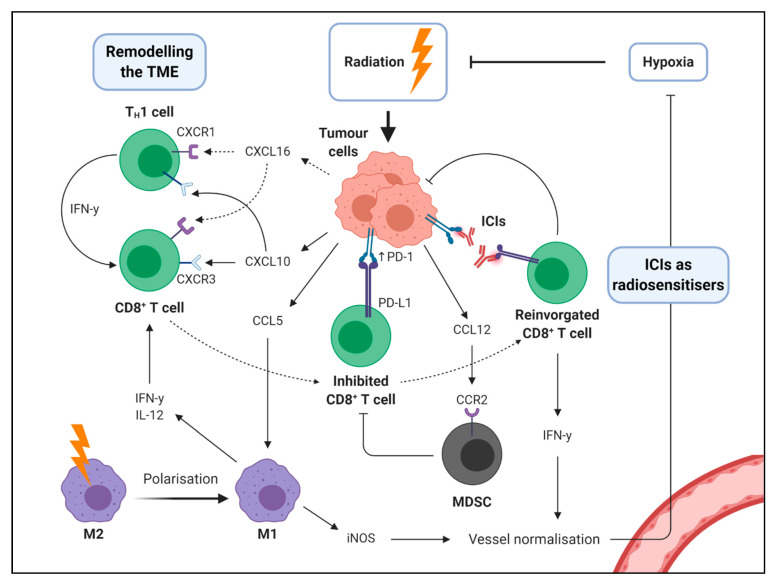
Radiation therapy can shape the immune response just as immunotherapy can shape the response to radiation therapy. Radiation induces production of a variety of chemokines that can facilitate and antagonise the anti-tumour response: T cells and M1 macrophages contribute to tumour elimination and can be recruited by CXCL16 or CXL10, and CCL5, respectively; myeloid-derived suppressor cells (MDSCs) are immunosuppressive and can be recruited by radiation-induced expression of CCL12. Immune checkpoint inhibitors (ICIs) boost anti-tumour immunity by disrupting suppressive signalling molecules such as PD-1, PD-L1 and CTLA-4 (latter not shown). By recruiting T cells (via aforementioned mechanisms) radiation therapy can augment this phenomenon. Furthermore, cytokines released by immune cells including IFN-γ from CD8^+^ T cells can promote tumour vessel normalisation counteracting hypoxia which promotes radioresistance: thus, by enhancing T cell responses ICIs can act as radiosensitisers.

However, these immunostimulatory aspects of radiotherapy can be counterbalanced by suppressive signalling. Regulatory T (T_reg_) cells are a therapeutic target for anti-tumour immunity; naturally occurring FoxP3^+^ T_reg_ cells supress immunity by direct cell to cell contact and inducible T_reg_ cells secrete TGF-β and IL-10 which promote immune escape [41]. These immunosuppressive elements are upregulated by radiotherapy, and their presence has been linked to poor response to ICIs in diverse patient cohorts [41,42]. Myeloid-derived suppressor cells (MDSCs: Gr1^+^CD11b^+^) are potent inhibitors of cytotoxic T cell function [43]. Radiotherapy can upregulate CCL2, which binds to the CCR2 receptor to promote MDSC accumulation in the TME [20]. Radiation also dampens effector T cell responses as a result of increased PD-L1 expression mediated by IFN-y [44]. Overall, this highlights a double-edged sword in the context of radiotherapy, representing an important mechanism of radioresistance while simultaneously promoting sensitivity to checkpoint blockade. As such, radiotherapy can have a dual effect on the TME, both enhancing the accumulation of effector and suppressive T cell and myeloid cell populations (Figure 3). 

### 2.6. Immunotherapy as a Radiosensitiser

Although the current focus of combining radiation and immunotherapy is to boost response to ICIs, emerging evidence indicates that immunotherapy itself is a potent radiosensitiser. The dysfunctional vasculature within tumours promotes radioresistance as a result of the hypoxic environment [43]. The lack of oxygen is responsible for the suppression of apoptosis during radiotherapy, as low oxygen availability limits ROS generation. Paradoxically, radiation itself can lead to the disruption of in vivo vascular systems around tumours, inducing a hypoxic response and activating hypoxia-inducible factor 1 (HIF1), thus reducing the generation of intratumoural ROS [45]. Hypoxia upregulates hypoxia inducible factor 1 (HIF-1), activating anaerobic glycolysis which produces lactate and antioxidants [46]. These antioxidants scavenge ROS, impeding radiation induced cell death, and lactate promotes immunotherapy resistance [47]. Recent data indicate that immunotherapy can normalise poorly formed, leaky hypoxia-promoting vessels in the TME. In preclinical models of breast and colon cancer, anti-PD-1 and anti-CTLA-4 therapy resulted in tumour regression, increased perfusion and reduced tumour hypoxia [48]. This vessel normalisation was mediated by IFN-γ producing CD8^+^ T cells. The angiostatic effects of IFN-γ may be related to reduced αVβ3 integrin dependent endothelial cell activation and survival [49], and was correlated with preclinical efficacy of anti PD-1 therapy [48,49]. Another in silico analysis found an association between immune-stimulating gene pathways (including *Ackr1*, *Il1r1*, *Il6st* and *Socs2*) and vessel normalisation related genes such as decreased expression of *Vegfa* and increased expression of *Angpt1/Angpt2* [50]. In response to ICIs, TH_1_ cells produce IFN-γ to normalise vessels and reduce hypoxia through increased pericyte coverage, decreased leakiness and decreased hypoxia. This suggests that ICIs remodel the tumour vasculature, augmenting their own efficacy and potentially acting as a radiosensitiser.

## 3. Clinical Trials of Radiation and Immunotherapy in UGI Cancer

### 3.1. Single Agent Immunotherapy

Single agent ICI trials in UGI cancers have delivered modest results. The ATTRACTION-2 phase III trial found that nivolumab (anti-PD-1) improved overall survival (OS; 5.3 vs. 4.1 months in the placebo group, *p* < 0.0001) in heavily pretreated GC or gastroesophageal junction cancer (GEJC) regardless of PD-L1 expression [51]. The KEYNOTE-059 phase II study evaluated pembrolizumab (anti-PD-1) versus chemotherapy in previously treated GC or GEJC, with the objective response rate (ORR) of 11.6%, with a longer median duration of response in PD-L1+ patients (16.3 vs. 6.9 months) [52]. Based on these results, in 2018 the FDA granted accelerated approval to pembrolizumab in the third line treatment of recurrent GC or GEJC that overexpresses PD-L1 with a Combined Positive Score [CPS] ≥ 10), as determined by a U.S. Food and Drug Administration (FDA)-approved test, with disease progression after one or more prior lines of systemic therapy as identified in KEYNOTE-181. Clinical efficacy of ICIs in OSCC is slightly more encouraging, with KEYNOTE 181, a phase III trial reporting that as second line treatment, the median OS with pembrolizumab vs. chemotherapy was similar in the intention to treat(ITT) group (7.1 vs. 7.1 months) and longer in the SCC (8.2 vs. 7.1 months) and PD-L1 CPS ≥10 groups (9.3 vs. 6.7 months) [53]. A trend was observed favouring responses in OSCC, forming the basis for the 2019 FDA approval of pembrolizumab in the second line treatment of metastatic PD-L1^+^ OSCC. More recently, the ATTRACTION-3 phase III trial of second line nivolumab vs. chemotherapy confirmed this OS benefit (10.9 months vs. 8.4 months, *p* = 0.019), further supporting the place of PD-1 inhibition in metastatic OSCC [54]. Approved indications of immunotherapy in gastroesophageal cancer are therefore confined to the second- or third-line treatment of metastatic disease. However, more recent data from the CheckMate-649 and KEYNOTE-590 studies indicate that the addition of nivolumab (median OS: 14.4 vs. 11.1, HR 0.71, *p* < 0.0001) or pembrolizumab (median OS: 12.4 vs. 9.8, HR 0.73, *p* < 0.0001) to first line chemotherapy in metastatic UGI cancers can prolong overall survival [55,56]. This suggests that combining ICIs with more traditional treatment modalities can improve outcomes in gastroesophageal cancers.

Prospective clinical data of combining immunotherapy and radiation are in a nascent phase; phase III trials have not been published outside of prostate and non small cell lung cancer (NSCLC). The phase III PACIFIC trial investigated durvalumab (anti PD-L1) following chemoradiotherapy in locally advanced NSCLC [57]. Compared to placebo, durvalumab improved overall survival (28.3 vs. 16.2 months) and both arms had similar rates of treatment related adverse events. A phase III trial in metastatic castration resistant prostate cancer found no benefit of ipilimumab (anti CTLA-4) following a single 8 Gy dose of radiation to up to five bone metastases [58]. In gastroesophageal cancers, trials are in early stages and the majority are ongoing. Clinical investigation focuses on three settings: disease treatable by surgical resection, definitive chemoradiotherapy and palliative treatment in the metastatic setting with timing of delivery and ideal combination multimodal therapies yet to be elucidated.

### 3.2. Locally Advanced Disease

Locally advanced, nonmetastatic UGI cancers are optimally treated by surgical resection. This is accompanied by the CROSS neoadjuvant chemoradiotherapy regimen consisting of carboplatin, paclitaxel and 41.4 Gy external beam radiotherapy (EBRT) [59]. However, only 15% of patients display a pathologic complete response (pCR) and trials are investigating if the addition of ICIs can improve response rates and patient outcomes (Table 1). One example is phase I trial (NCT03044613) of pembrolizumab (anti PD-1) in combination with the CROSS regimen in stage II/II OAC, OSCC or GEJC. Primary endpoints are pCR rate and treatment related adverse events (TRAEs). Preliminary results for the first 10 patients show an encouraging pCR rate of 40%, with acceptable toxicity and no delays in surgery [60]. A larger (n = 28) phase II trial of pembrolizumab in OSCC has reported a pCR rate of 46.1%, with 82% of patients surviving at 12 months [61]. However, 2/28 patients did not undergo surgery and two treatment related deaths were reported due to acute lung injury emphasising the need for TRAE monitoring. Another approach is the use of adjuvant ICIs in postoperative patients to better control micro-metastatic disease. A phase II trial has evaluated durvalumab (anti PD-L1) in OAC or GEJC previously treated with external beam radiotherapy (EBRT) with residual disease following tri-modality treatment of surgery and chemoradiation (NCT02639065). Early results indicate that adjuvant durvalumab is safe with few dose limiting toxicities [62]. Relapse free survival in this single arm study was 79.2%, which compares favourably to the historical rate of 50%. These encouraging results further underscore the need for more data in an adjuvant setting.

### 3.3. Definitive Chemoradiotherapy

Definitive chemoradiotherapy (dCRT) is an alternative standard of care in OSCC and is also employed in localised OAC deemed unsuitable for surgery. A 50.4 Gy EBRT is delivered in 25 fractions, accompanied by 5-FU and cisplatin or FOLFOX (5FU, folinic acid and oxaliplatin). dCRT may promote immunogenic changes in tumours, priming tumours for ICI treatment to further enhance local and distant tumour control. The randomised, doubled blinded, phase III KEYNOTE-975 trial is evaluating pembrolizumab with traditional dCRT in localised but inoperable OSCC, OAC and GEJC (NCT04210115). The primary endpoints are overall survival and event free survival, and results could define a new standard of care in the dCRT setting. Other ongoing trials are evaluating dual checkpoint blockade (anti PD-1 and anti CTLA-4; NCT03437200), and sequential nivolumab and cetuximab (anti EGFR) with concomitant dCRT.

### 3.4. Systemic Treatment of Advanced Disease

Combining ICIs and radiation in the recurrent or metastatic setting seeks to activate the abscopal response, priming antigen specific CD8^+^ T cells against tumours outside the radiation field. The few radio-immunotherapy trials in metastatic UGI cancers are at an early stage and seek to investigate toxicities and mechanisms of response. One example, phase II trial of pembrolizumab and 30 Gy conventional fractionated radiotherapy is recruiting patients with metastatic gastroesophageal cancers (NCT03544736). Primary endpoints aim to quantify the abscopal response; changes in CD8^+^ TILs at the irradiated site, and changes in MDSCs and Tregs at peripheral metastases will be measured. Another approach is a combination of nivolumab and high dose brachytherapy to deliver 16 Gy over 2 fractions (NCT02642809), and durvalumab with concomitant chemoradiotherapy in the palliative setting (NCT03544736).

## 4. Optimising Radiation Parameters within Immunotherapy

### 4.1. Radiation Dose

Conventional fractionated radiation is delivered in small 1.8–2 Gray (Gy) daily fractions. For example, the CROSS regimen for OAC and OSCC involves 41.4 Gy given in 23 fractions of 1.8 Gy each, five days per week [59]. Recent advances in radiation technique and delivery, including intensity modulated radiotherapy (IMRT), volumetric-modulated arc therapy (VMAT) and proton beam therapy allow delivery of higher radiation doses while minimising acute and long-term toxicity. This has allowed a shift to ‘hypo-fractionated’ approaches, ranging from 5–10 Gy over three to five fractions to single doses of up to 24 Gy using stereotactic body radiotherapy (SBRT). SBRT employs advanced imaging, immobilisation techniques and real-time organ motion tracking, used to ablate oligometastatic disease. Hypofractionated radiotherapy can minimise toxicity while maintaining efficacy, favoured in oesophageal cancer patients unfit to receive definitive chemoradiation therapy [60].

**Figure 4:**
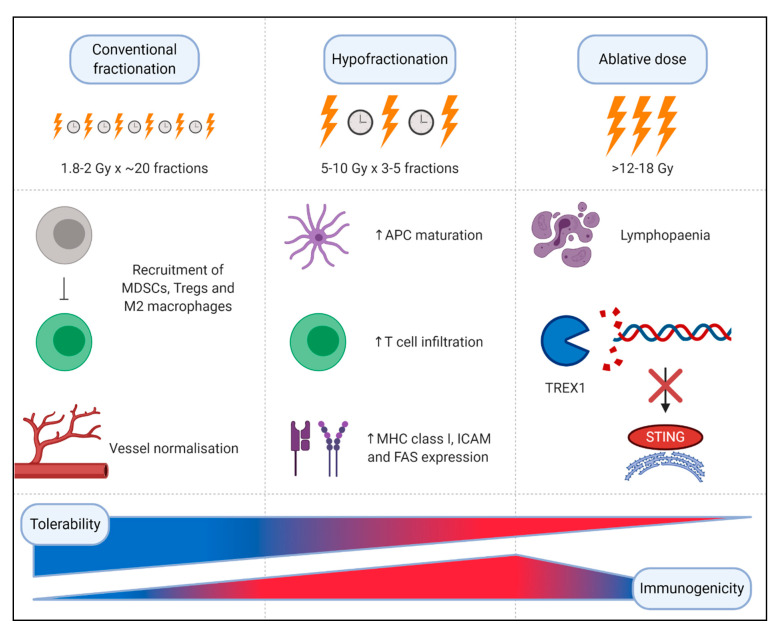
Optimising radiation dose and delivery for optimal immunogenicity. Small doses of radiation delivered in conventional fractionated radiotherapy are believed to have immunosuppressive effects in the tumour microenvironment; accumulation of immunosuppressive cell types such as myeloid-derived suppressor cells (MDSCs), regulatory T cells (Tregs) and M2 macrophages repress anti-tumour immunity. This immunosuppressive effect is somewhat counterbalanced by the normalising effect low dose radiation has on the tumour vasculature. Higher doses of radiation, such as those used in hypofractionation, can have a stimulatory immunogenic effect mediated by increased antigen presenting cell (APC) maturation, in addition to augmented T cell infiltration and enhanced expression of immunogenic proteins including MHC class I, ICAM and FAS on tumour cells. However, once the dose of radiation surpasses 12–18 Gy, immunogenicity is compromised: TREX1, an exonuclease, is induced leading to degradation of cytoplasmic DNA, thus negatively regulating the cGAS-STING pathway.

It is postulated that conventional fractionation can have immunosuppressive effects in the TME by recruiting MDSCs, Treg cells and M2-macrophages, potentially mediated by TGF-β upregulation [61]. However, this conventional fractionation can also have the immunogenic effect of normalising the tumour vasculature [39]. Higher radiation doses per fraction (>6 Gy) have more profound immunological effects, including facilitating maturation of APCs, increasing T cell infiltration, enhancing MHC-I expression and tumour peptide presentation and upregulation of immunostimulatory signals like Fas and ICAM on tumour cells [61,62]. However, the immunogenic effects of high dose radiation seem to have a limit; higher ablative doses (>12–18 Gy) induce *TREX1*, an exonuclease that degrades cytoplasmic DNA [63]. This negatively regulates the cGAS-STING pathway that is vital in radiation-induced immunogenicity. For this reason, hypofractionated radiotherapy (e.g., 3 × 8 Gy doses) has the most potential as a favourable immunomodulator [63] (Figure 4).

### 4.2. Radiation Timing

The immunological effects of radiation are time-dependent. In vitro there is an increase in the MHC-I peptide pool after 8 h, lasting for 11 days [31]. Clinical samples show increased activated dendritic cells during the first week, potentially correlating with radiation-induced antigen presentation [64]. Populations of activated and proliferating T cells declined in the first week but increased after the third week of therapy, supporting radiation as an in-situ vaccine [64]. These time-dependent effects can be exploited in the clinic. Multivariable analysis in the PACIFIC trial found that ICI initiation <2 weeks following chemoradiotherapy was associated with greater overall survival [57]. This survival benefit when ICI is initiated concurrently or shortly after radiation was also found in a retrospective analysis of 750 patients [65]. This suggests that radiation may ‘prime’ the tumour for optimal immunotherapy efficacy, a principle that could be employed to maximise ICI efficacy in gastroesophageal cancers.

### 4.3. Radiation Adverse Effects

Radiotherapy is associated with a host of adverse effects, but radiation-induced lymphopenia (RIL) is most relevant to ICI treatment. Lymphocytes are critical for the anti-tumour immune response, and T cell-deficient mice are unable to mount abscopal responses [66]. Indeed, RIL is an independent predictor of poor overall survival in UGI cancers [67]. In addition to combination chemotherapy, the radiotherapy target volume is a key determining factor in incidence of RIL. This is due to radiation doses to sites of lymphopoiesis (bone marrow) or lymphocyte storage (spleen, lymph nodes) [61]. Advances in fractionation may ameliorate this adverse effect; in two pancreatic cancer cohorts, hypofractionated radiotherapy delivered by SBRT was associated with less RIL when compared to standard fractionation (1.8 Gy), highlighting that hypofractionated approaches may also have favourable toxicity as well as efficacy [68,69]. As well as direct depletion of lymphocytes, elective irradiation of draining lymph nodes has additional detrimental effects [70]. Compared to irradiating the primary tumour alone, elective nodal irradiation is associated with altered intratumuoral chemokine expression and CD8^+^ T cell trafficking, as this was correlated with poorer survival in a combination of radiation and immunotherapy [70]. Immunotherapy carries a risk of immune-related adverse events (irAEs), including a potentially life-threatening pneumonitis. Radiation-induced lung injury is defined by pneumonitis and fibrosis and is a common dose limiting toxicity of radiotherapy in UGI cancers. Therefore, a combination of radiation and immunotherapy could increase incidence and severity this adverse effect [61]. The KEYNOTE-001 reported a higher rate of ICI-related pneumonitis in those that had previously received thoracic radiation, and several case reports have been published of severe pneumonitis in patients treated with ICI and SBRT [71]. In the PACIFIC trial, both the durvalumab and placebo arms had similar incidence of pneumonitis [57], so further data are needed to achieve a balance between safety and efficacy in UGI cancers.

### 4.4. Future Directions

Despite the success in leveraging the combination of immunotherapy for the treatment of upper gastrointestinal cancers, several issues are outstanding. There is a lack of studies that directly compare the immunogenicity of different dosing and fractionation regimens. This is seen in both preclinical studies and clinical trials, and not specific to gastrointestinal cancers. Some studies vary the entire dose of radiation but do not ascertain whether this dose would be better delivered in a single ablative dose, a hypofractionated regimen or conventional 1.8–2 Gy fractionation. Well-controlled preclinical studies would provide more clarity on the subtle effects of different fractionation, while a multi-arm clinical trial would be useful to determine the optimal dosage regimen for clinical practice. These trials should also have translational study endpoints including abscopal response and changes in CD8^+^ TILs, T_reg_ cells, and MDSCs levels in the irradiated and peripheral sites. This would be a means of dissecting the mechanisms of action and resistance to immuno-radiotherapy, and provide mechanistic data specific to upper GI cancers. Finally, there is a need for more trials in advanced disease setting. In contrast to single agent ICB, most trials of ICB and radiotherapy are in locally advanced, resectable disease where chemoradiotherapy is a standard of care. However, outcomes are worst in the refractory disease setting and response rates to single agent ICB is low [72]. This highlights an unmet need for trials in this population, which could stand to benefit most of this symbiotic combination.

## 5. Conclusions

The advent of immune checkpoint blockade has shifted the paradigm in the treatment of solid tumours, but the impact of ICIs on patient outcomes in UGI cancers has been limited. Radiotherapy has the potential to augment responses to ICI through cGAS-STING signalling, immunogenic cell death, upregulation of neoantigen expression and through inflammatory remodelling of the immune microenvironment. Given the extensive pre-existing use of radiation and the modest activity of single agent immunotherapy, gastroesophageal cancers are poised to greatly benefit from a combination of ICIs and radiotherapy. However, questions remain surrounding methods of optimising the radiation dose and timing while minimising toxicity. Most ongoing clinical trials employ conventional radiation fractionation, although preclinical data suggest that hypofractionated regimens are favourable in terms of toxicity and efficacy. A better understanding of variability in response to immune checkpoint blockade is also required. Future trials should incorporate correlative endpoints to identify predictive biomarkers of response as this will help to select patients likely to benefit from radiation and immunotherapy and facilitate a precision oncology approach.

## Figures and Tables

**Table 1:** Ongoing clinical Trials of radiation and immunotherapy in operable disease.

Identifier	Phase	N	Disease Setting	Treatment	Radiation	Primary Endpoint
NCT03792347	I	20	Stage II/III OSCC	Pembrolizumab, carboplatin, and paclitaxel	41.4 Gy in 23 fractions	TRAEs
NCT02844075	II	18	Stage II/III OSCC	Pembrolizumab, carboplatin, and paclitaxel	41.4 Gy in 23 fractions	pCR rate
NCT03257163	II	40	Stage II/III dMMR or EBV^+^ GC	Neoadjuvant pembrolizumab	Conventional Fractionation	RFS
				Adjuvant Capecitabine and pembrolizumab		
NCT03064490	II	38	Stage II/III GC or OAC	Pembrolizumab, carboplatin, and paclitaxel	41.4 Gy in 23 fractions	pCR rate
NCT02730546	I/II	68	Stage II/III GC or GEJC	Pembrolizumab, carboplatin, and paclitaxel	41.4 Gy in 23 fractions	pCR rate
						PFS
NCT03044613	I	25	Stage II/III OAC, OSCC or GEJC	Nivolumab and carboplatin and paclitaxel	41.4 Gy in 23 fractions	TRAEs
NCT03776487	I/II	30	Stage II/III GC or GEJC	FOLFOX and Nivolumab and ipilimumab followed by surgical resection	50 Gy 25 fractions	TRAEs
NCT02962063	II	35	Stage II/III GEJC and GC	Neoadjuvant Durvalumab and mFOLFOX Adjuvant durvalumab	50 Gy in 28 fractions	TRAEs
						pCR rate
NCT04159974	II	56	Stage II/III OAC or GEJC	Durvalumab, carboplatin and paclitaxel	41.4 Gy in 23 fractions	pCR rate
						TRAEs
NCT02639065	II	23	Stage II/III OAC or GEJC with residual disease	Durvalumab	41.4 Gy in 23 fractions	TRAEs
						DLTs
NCT03490292	I/II	24	Stage II/III OSCC or OAC	Avelumab and Carboplatin, paclitaxel	41.4 Gy in 23 fractions	DLT
						pCR

Abbreviations: TRAEs, treatment related adverse effects; pCR, pathological complete response; DLT, dose limiting toxicity; OAC, oesophageal adenocarcinoma; OSCC, oesophageal squamous cell carcinoma; GEJC, gastroesophageal junction adenocarcinoma; dMMR, deficient mismatch repair; Gy, Gray; RFS, relapse free survival.
